# Correction: Synthesis of DNA-guided silver nanoparticles on a graphene oxide surface: enhancing the antibacterial effect and the wound healing activity

**DOI:** 10.1039/c9ra90087j

**Published:** 2019-11-29

**Authors:** Chunyi Tong, Wei Zou, Weimin Ning, Jialong Fan, Li Li, Bin Liu, Xuanming Liu

**Affiliations:** College of Biology, Hunan Province Key Laboratory of Plant Functional Genomics and Developmental Regulation, Hunan University Changsha 410082 PR China binliu2001@hotmail.com +86 731 89720939 +86 731 89720939; Key Laboratory of Hunan Provincial TCM Administration for TCM in Obstetrics & Gynecology, Hunan Provincial Maternal and Child Health Care Hospital Changsha 410008 PR China

## Abstract

Correction for ‘Synthesis of DNA-guided silver nanoparticles on a graphene oxide surface: enhancing the antibacterial effect and the wound healing activity’ by Chunyi Tong *et al.*, *RSC Adv.*, 2018, **8**, 28238–28248.

In the published article in Fig. 6E, the enlarged pictures of GO and ssDNA-AgNP groups were duplicated, and the corrected version is shown below.

**Fig. 6:**
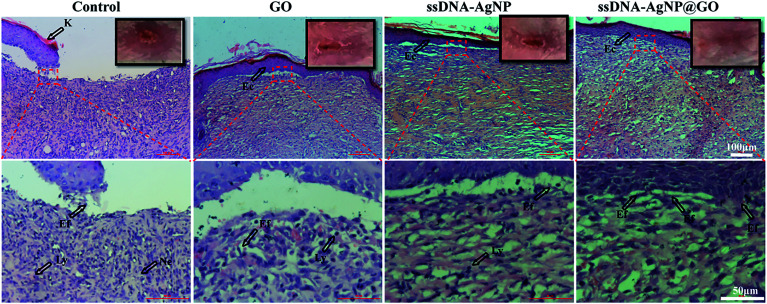
(E) Histology of the wound healing process in various groups on 14 days with H&E staining at magnifications of ×10 and ×40. Lines indicate wound healing events. K = keratin, Ly = lymphocyte, Ne = neutrophil, Ec = epithelial cells and Ef = elongated fibroblasts. The presence of Ly and Ne indicate an inflammatory response. Ec and Ef were the signals of re-epithelization, which is beneficial for the formation of matured fibrous granulation tissue.

Additionally, in Fig. S7 (ESI), kidney slice pictures of control and GO groups and lung slice pictures of ssDNA-AgNPs and ssDNA-AgNPs@GO were duplicated. A revised version of the ESI has been published.

The Royal Society of Chemistry apologises for these errors and any consequent inconvenience to authors and readers.

## Supplementary Material

